# Mechanisms Underlying Drought Adaptability in Duolang Sheep Based on Metabolomic and Transcriptomic Analyses

**DOI:** 10.3390/biology15060461

**Published:** 2026-03-12

**Authors:** Bingjie Jiang, Bin Chen, Yaling Yang, Yong Chen, Wujun Liu

**Affiliations:** Department of Animal Science, Xinjiang Agricultural University, Urumqi 830052, China

**Keywords:** Duolang sheep, drought adaptability, immune response, lipid metabolism

## Abstract

This study investigates the mechanisms underlying drought adaptability in Duolang sheep, a local breed from two distinct habitats in Xinjiang—an arid southern region and a grassland northern region—aiming to identify key factors driving differential environmental adaptation. Integrated multi-omics analyses were performed, including serum biochemical assays, untargeted metabolomics of perirenal and tail fat tissues, and transcriptomic profiling of lung, liver, and kidney samples. Our results revealed notable differences: (1) serum levels of GSH-Px, IL-2, and IgG were significantly higher in the southern group (*p* < 0.01); (2) metabolomic analysis identified key differential metabolites, including EPA (involved in unsaturated fatty acid biosynthesis), choline (glycerophospholipid metabolism), L-serine and glutathione (cofactor biosynthesis), and taurine (sulfur metabolism); and (3) transcriptomic analysis revealed significant differential expression of genes such as FGF21 (thermogenesis), CD14 and DUSP2 (MAPK signaling pathway), GOT1 (arginine biosynthesis), and AVPR2 (vasopressin-regulated water reabsorption). Integrative correlation analysis further indicated that glutathione, EPA, GOT1, and CD14 are involved in energy and lipid metabolism, while taurine, AVPR2, and DUSP2 contribute to oxidative stress resistance and immune regulation. These molecular and metabolic adjustments collectively enhance drought adaptability in southern Xinjiang Duolang sheep. In conclusion, adaptation to arid environments requires enhanced antioxidant capacity and immune function, with metabolites such as EPA supporting lipid metabolism and genes such as FGF21 regulating fatty acid oxidation to limit triglyceride accumulation.

## Simple Summary

This study investigates the drought adaptability of Duolang Sheep, with the goal of identifying key regulatory factors involved in this trait. To achieve this, we conducted integrated analyses using serum biochemical assays, untargeted metabolomics, and eukaryotic reference-based transcriptomics. Our findings provide a theoretical foundation for identifying resistance-related genes in Duolang Sheep under arid conditions and offer valuable insights for the genetic improvement of this breed.

## 1. Introduction

Animal adaptation to diverse environments plays a fundamental role in shaping biodiversity and driving species evolution. Investigating the underlying mechanisms of environmental adaptation provides a critical theoretical foundation for livestock management and the selective breeding of stress-resilient animal populations. Environmental adaptability refers to the capacity of animals to survive, reproduce, maintain health, and sustain productivity under challenging conditions, including high temperatures, humidity, limited feed availability, and pathogen exposure. As a complex trait, it is closely associated with thermotolerance, disease resistance, stress responsiveness, and metabolic efficiency [1].

Xinjiang, located in northwestern China, is a typical arid and semi-arid region and accounts for one-sixth of the country’s total land area [2]. The Kashgar region, situated within the Tarim River Basin, represents a characteristic arid zone ecosystem in China [3]; Aksu Prefecture, also part of the Tarim Basin, shares a similar ecological environment. This area falls within a warm temperate arid climate zone and lies on the northern margin of the Tarim Basin. It is marked by low precipitation, high evaporation, dry conditions, and abundant solar and thermal resources. Annual sunshine duration ranges from 2800 to 3000 h and the frost-free period lasts between 200 and 220 days, supporting the integrated development of agriculture, forestry, animal husbandry, and fishery. The average annual precipitation is approximately 75 mm, while annual evaporation reaches 1200 to 1500 mm. The mean annual temperature hovers around 10 °C. By contrast, Urumqi experiences a temperate continental arid climate, with brief spring and autumn seasons, extended winters and summers, and marked diurnal temperature variation. Its average annual precipitation is about 294 mm. Duolang sheep are a high-quality local dual-purpose breed raised for both meat and fat in the Kashgar region of Xinjiang. Known for their tolerance to roughage and strong stress resistance, Duolang sheep produce tender, juicy meat with even fat deposition and no mutton odor. The breed also exhibits high meat yield and strong fecundity [4]. As fat-tailed sheep with abundant tail fat, they serve as ideal models for studying adipogenesis, differentiation, and fat deposition mechanisms.

Studies have demonstrated that indigenous sheep in Xinjiang adapt to extreme arid environments by enhancing antioxidant capacity to reduce water loss and improving renal water reabsorption function. Similarly, Egyptian fat-tailed sheep have evolved metabolic adaptations that increase gluconeogenesis and fatty acid metabolism, thereby improving energy utilization efficiency and reducing water consumption. These findings suggest that sheep employ synergistic physiological strategies at multiple levels to build resilience against arid conditions, highlighting their considerable adaptive capacity [5]. It is well established that arid environments impose multifaceted physiological challenges: they elevate oxidative stress levels and increase free radical production. In response, antioxidant metabolites such as glutathione [6] and L-serine [7] play critical roles in mitigating oxidative damage.

To date, numerous studies have employed RNA-seq technology to investigate adaptive changes in animals under arid conditions. Camels, often referred to as the “ships of the desert,” exhibit remarkable adaptability to extreme drought. Transcriptomic analyses have revealed significant gene expression changes in response to arid stress. For instance, [8] conducted transcriptomic profiling of liver and kidney tissues in dromedary camels and found that genes involved in water retention and salt balance—such as aquaporins and salt transporters—were significantly upregulated. These expression adjustments help camels maintain fluid homeostasis in water-scarce environments. In a related study, reference [9] contains comparative genomic and transcriptomic analyses of dromedary camels, Bactrian camels, and alpacas. Their work uncovered genetic features associated with water and fat metabolism, as well as responses to heat, drought, ultraviolet radiation, and dust exposure, and provided insights into the evolutionary adaptation of the kidney to desert conditions.

In this study, we measured serum biochemical indices in Duolang sheep from two distinct regions: southern Xinjiang (arid environment) and northern Xinjiang (relatively humid environment). We performed untargeted metabolomic analysis on two adipose depots—perirenal and caudal fat—and conducted eukaryotic reference-based transcriptomic (RNA-seq) analysis on lung, liver, and kidney tissues. As an important high-fecundity sheep germplasm resource in China, Duolang sheep play a key role in local economic development and breed improvement. However, research on the mechanisms underlying their adaptability to arid environments remains limited. This study aims to identify key biological changes associated with arid adaptation in Duolang sheep through integrated multi-omics analyses, thereby revealing the potential mechanisms of their environmental adaptability. These findings are expected to provide a theoretical foundation for mining resistance genes and offer insights for the genetic improvement of Duolang sheep under arid conditions.

## 2. Materials and Methods

### 2.1. Experimental Animals and Sample Collection

This experiment was conducted in Aksu and Urumqi between December 2024 and January 2025. A total of 30 healthy, one-year-old male Duolang sheep were selected, with 15 individuals from southern Xinjiang (Aksu) and 15 from northern Xinjiang (Urumqi). (During the actual sample measurement process, only 12 samples of Northern Xinjiang Duolang sheep met the standards, while all 15 samples of Southern Xinjiang Duolang sheep were deemed suitable and subsequently used for transcriptome and metabolome sequencing, i.e., *n* = 15 for the treatment group, *n* = 12 for the control group). All animals were raised under consistent feeding and management conditions. Prior to slaughter, the sheep were fasted for 24 h and deprived of water for 2 h, after which body weights were recorded. Following blood collection, approximately 100 g of caudal fat, perirenal fat, lung, liver, and kidney tissues were harvested from each animal. Two portions of each tissue sample were preserved for subsequent sequencing analyses.

### 2.2. Determination of Serum Indicators

All serum samples were analyzed for biochemical indicators using ELISA kits (Nanjing Jiancheng Bioengineering Institute, Nanjing, China) according to the manufacturer’s instructions. The measured parameters included biochemical markers such as glucose, total protein, albumin, total cholesterol, triglycerides, lactate dehydrogenase, alanine transaminase, and aspartate transaminase, as well as antioxidant indices including total antioxidant capacity, glutathione peroxidase, superoxide dismutase, and malondialdehyde. Detailed assay protocols are provided in Table A1.

### 2.3. Untargeted Metabolomics Analysis

#### 2.3.1. Metabolite Extraction and Detection

A 20 mg (±1 mg) tissue sample was accurately weighed into a labeled centrifuge tube. Then, 400 μL of 70% methanol aqueous solution containing internal standards was added, and the mixture was vortexed for 3 min. Following ultrasonication in an ice-water bath for 10 min, the sample was vortexed again for 1 min and incubated at −20 °C for 30 min. After centrifugation at 12,000 rpm for 10 min at 4 °C, 300 μL of the supernatant was transferred to a new labeled centrifuge tube. A second centrifugation was performed at 12,000 rpm for 3 min at 4 °C, and 200 μL of the resulting supernatant was transferred to a sample vial insert for subsequent instrumental analysis.

#### 2.3.2. Untargeted Metabolomic Analysis

The supernatant was transferred to amber sample vials for LC-MS/MS analysis. Chromatographic separation was performed on a Waters ACQUITY Premier HSS T3 Column (1.8 µm, 2.1 mm × 100 mm) coupled to a Q Exactive HF-X mass spectrometer (Yeasen Biotechnology (Shanghai) Co., Ltd., Shanghai, China). The mobile phase consisted of solvent A (0.1% formic acid in water) and solvent B (0.1% formic acid in acetonitrile). Mass spectrometry parameters were set as follows: spray voltage of 3500 V in positive ion mode and −3200 V in negative ion mode; capillary temperature at 320 °C; sheath gas flow rate at 30 arbitrary units (A.U.); and auxiliary gas flow rate at 5 A.U. Both full-scan MS and MS/MS data were acquired using a Vanquish ultra-high performance liquid chromatography (UHPLC) system.

### 2.4. Eukaryotic Reference-Based Transcriptomic (RNA-Seq) Analysis

#### 2.4.1. Total RNA Extraction and Quality Control

Total RNA was extracted from liver, lung, and kidney tissues of Duolang sheep using TRIzol reagent (see Table A1 for detailed procedures). RNA concentration and purity were assessed using a NanoDrop 2000 spectrophotometer (Thermo Fisher Scientific, Wilmington, DE, USA), and RNA integrity (RIN) was evaluated with an Agilent 2100 Bioanalyzer (Agilent Technologies, Santa Clara, CA, USA). Only RNA samples with RIN > 8.5 and a 28S/18S ratio > 0.7 were used for subsequent library construction.

#### 2.4.2. Library Construction and Sequencing

Total RNA was treated with DNase I to remove genomic DNA contamination. mRNA was then enriched using Oligo(dT)-conjugated magnetic beads, fragmented into short segments, and used as a template for double-stranded cDNA synthesis. The resulting cDNA was purified, followed by end repair, A-tailing, and adapter ligation. After fragment size selection, the cDNA library was enriched via PCR and validated for concentration and insert size (effective concentration > 2 nM). Paired-end sequencing (PE150) was performed on an Illumina NovaSeq 6000 platform (Beijing Biomarker Technologies Corporation, Beijing, China).

### 2.5. Statistical and Bioinformatic Analysis

Identified metabolites and differentially expressed genes in each comparison group were subjected to comprehensive functional annotation, including Gene Ontology (GO) enrichment analysis, Kyoto Encyclopedia of Genes and Genomes (KEGG) pathway analysis, and protein–protein interaction (PPI) network construction. Serum biochemical data were analyzed using an independent samples *t*-test in SPSS 27.0, with *p* < 0.05 considered statistically significant. Data integration and preliminary processing were performed using Microsoft Excel. The interaction network of key candidate genes was visualized with Cytoscape (Version 3.10.3).

## 3. Results

### 3.1. Analysis of Serum Biochemical Indicators

As shown in Table 1, comparative analysis of physiological and biochemical indicators between Duolang sheep from southern and northern Xinjiang revealed highly significant differences (*p* < 0.01) in the levels of glutathione peroxidase (GSH-PX, U/mL), interleukin-2 (IL-2, ng/L), and immunoglobulin G (IgG, mg/mL). Specifically, the levels of GSH-PX, IL-2, and IgG were significantly higher in sheep from southern Xinjiang than in those from northern Xinjiang (*p* < 0.01). No significant differences were observed in the remaining physiological and biochemical indicators measured.

### 3.2. Untargeted Metabolomics Analysis

**Figure 1:**
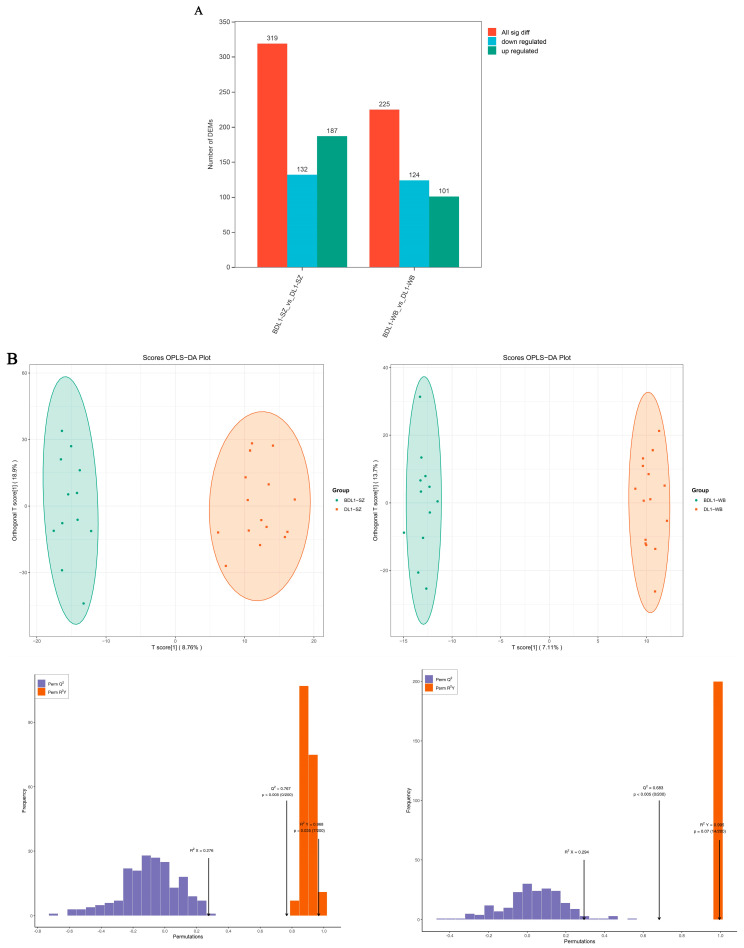

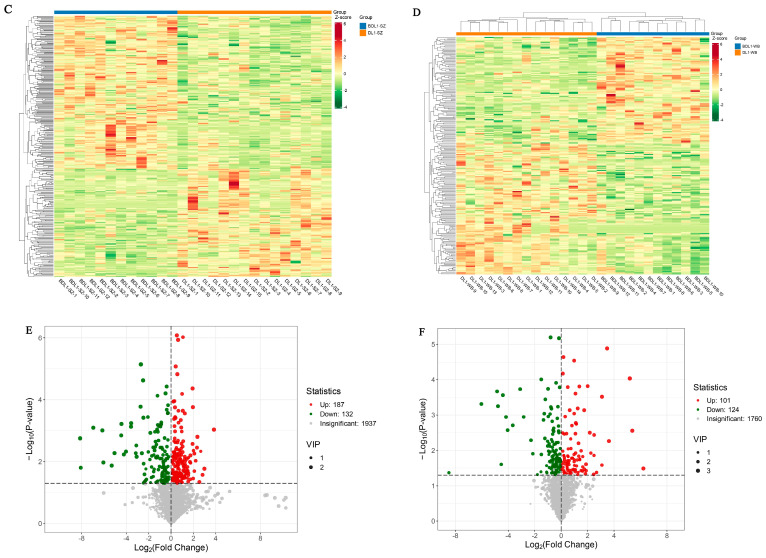
Expression profiles of differential metabolites in southern and northern Xinjiang Duolang sheep. Note: Expression profiles of differential metabolites in Duolang sheep from southern and northern Xinjiang. (**A**): Bar chart showing the comparison of the number of differential metabolites between the two groups. (**B**): OPLS-DA plot. (**C**,**D**): Clustered heatmaps of differential metabolites in different tissues. (**E**,**F**): Volcano plots of differential metabolites in different tissues. Up: Metabolites upregulated in Group 2 compared with Group 1; Down: Metabolites downregulated in Group 2 compared with Group 1. DL-SZ: Perirenal fat of Duolang sheep from southern Xinjiang; DL-WZ: Tail fat of Duolang sheep from southern Xinjiang; BDL-SZ: Perirenal fat of Duolang sheep from northern Xinjiang; BDL-WZ: Tail fat of Duolang sheep from northern Xinjiang; the same below.

Through the two comparisons of BDLSZ_vs_NDLSZ and BDLWZ_vs_NDWZ, 319 and 225 differential metabolites were identified, respectively (Figure 1A). The OPLS-DA model score plot (Figure 1B) showed that the two populations (southern and northern Xinjiang Duolang sheep) were significantly clustered in relatively tight regions, with significant differences in expression patterns between them. In the permutation test, Q^2^ > 0.5 was used to determine the presence of significant differences between the two groups. As shown in the figure, the Q^2^ values across all tissues were greater than 0.5, confirming that the model is valid and reliable. Analysis of the differential metabolite clustering heatmaps (Figure 1C,D) indicated that the BDLSZ_vs_NDLSZ and BDLWZ_vs_NDWZ groups exhibited significant differences, i.e., there were significant differences in differential metabolites between northern and southern Xinjiang Duolang sheep. As shown in the volcano plots, the BDLSZ_vs_NDLSZ group contained 187 upregulated differential metabolites and 132 downregulated differential metabolites; the BDLWZ_vs_NDWZ group contained 101 upregulated differential metabolites and 124 downregulated differential metabolites.

**Figure 2:**
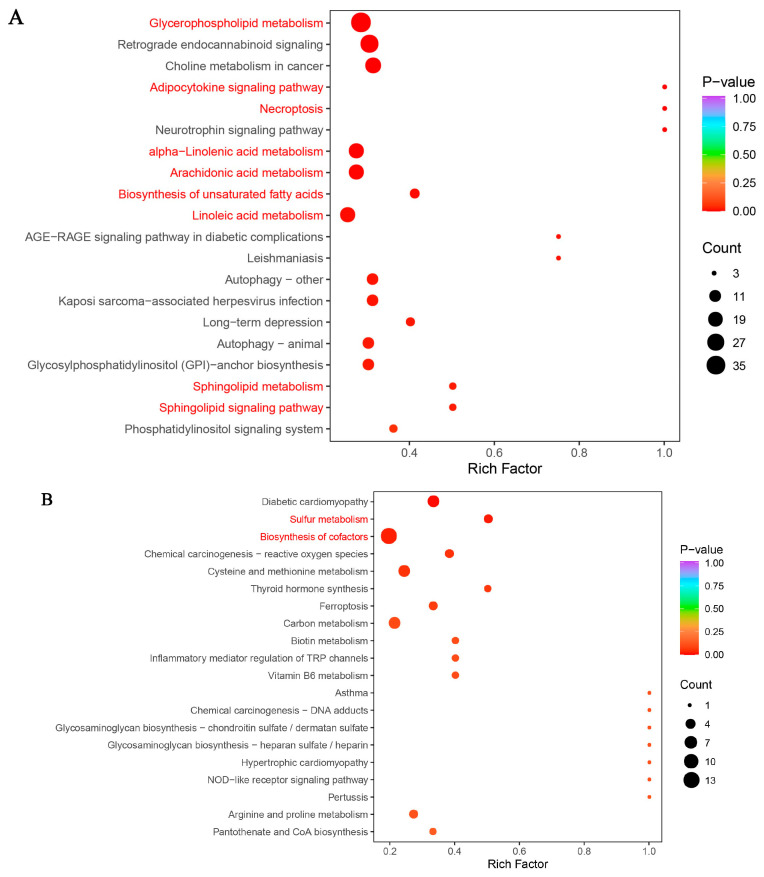

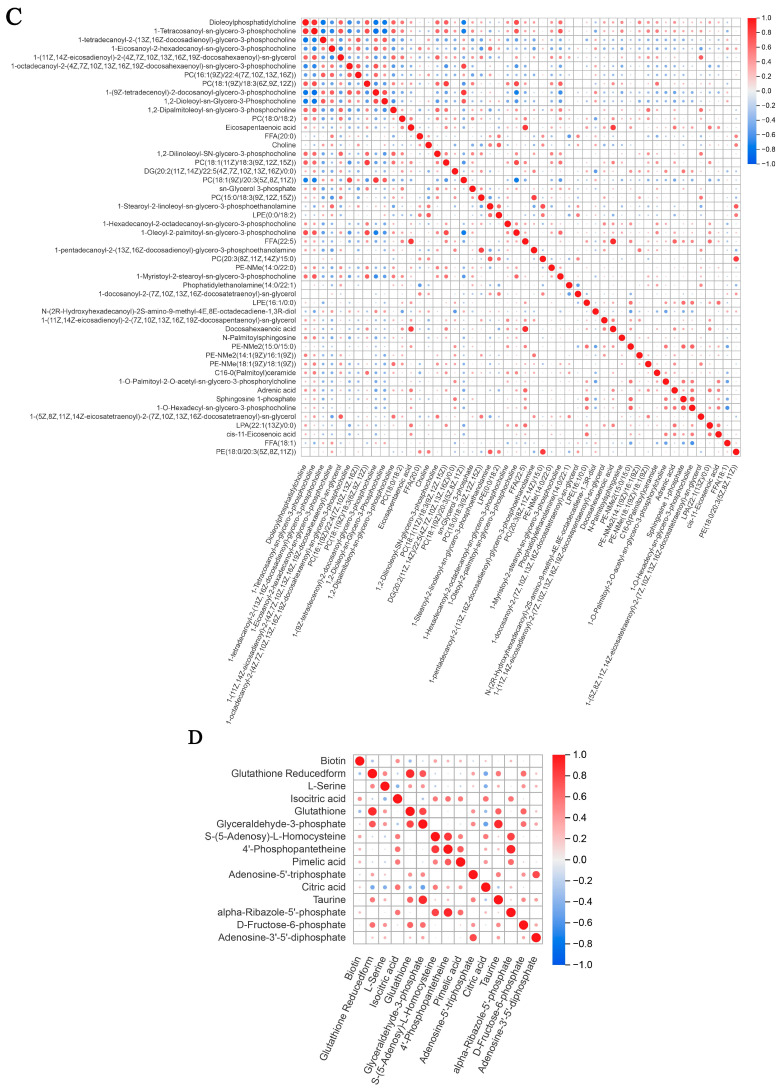
KEGG functional annotation analysis of differentially expressed metabolites among Duolang sheep from northern and southern Xinjiang. Note: (**A**,**B**): KEGG enrichment analysis plots in different tissues. In subfigures (**A**,**B**), the red text indicates the key candidate pathways that are significantly expressed in each part (*p* < 0.05). The same below. (**C**,**D**): Differential metabolite correlation matrix plots in different tissues. In subfigures (**C**,**D**), the varying sizes of the circles indicate the strength of the correlation, with red representing a positive correlation and blue representing a negative correlation. The same below.

KEGG enrichment analysis was performed on differential metabolites identified in the BDLSZ_vs_NDLSZ and BDLWB_vs_NDLWB groups (comparing Duolang sheep from northern and southern Xinjiang), with results presented in Figure 2. Key pathways were filtered using a threshold of *p* < 0.05, and the findings are summarized as follows: In the BDLSZ_vs_NDLSZ group, 51 differential metabolites were enriched in 19 KEGG pathways. Among these, six key candidate pathways were associated with metabolism: glycerophospholipid metabolism, sphingolipid metabolism, arachidonic acid metabolism, linoleic acid metabolism, alpha-linolenic acid metabolism, and biosynthesis of unsaturated fatty acids. One key candidate pathway was related to environmental information processing: the sphingolipid signaling pathway. Another was linked to cellular processes: necroptosis. Additionally, one key candidate pathway involved organismal systems: the adipocytokine signaling pathway.

To explore the interactions among differential metabolites, correlation network analysis was performed for each comparison group. Candidate key differential metabolites identified from the two adipose depots were visualized separately. Using a significance threshold of *p* < 0.05, the following key metabolites were identified: In the BDL-SZ vs. NDL-SZ group, eicosapentaenoic acid (EPA)—a downregulated metabolite—showed a strong positive correlation with FFA (20:0) and was therefore selected as a key candidate differential metabolite. In the BDL-WB vs. NDL-WB group, upregulated glutathione and reduced glutathione were significantly positively correlated, while upregulated taurine exhibited a significant positive correlation with pantetheine-4′-phosphate. Based on these correlations, glutathione and taurine were identified as key candidate differential metabolites.

### 3.3. Eukaryotic Reference-Based Transcriptomics (RNA-Seq) Analysis

**Figure 3:**
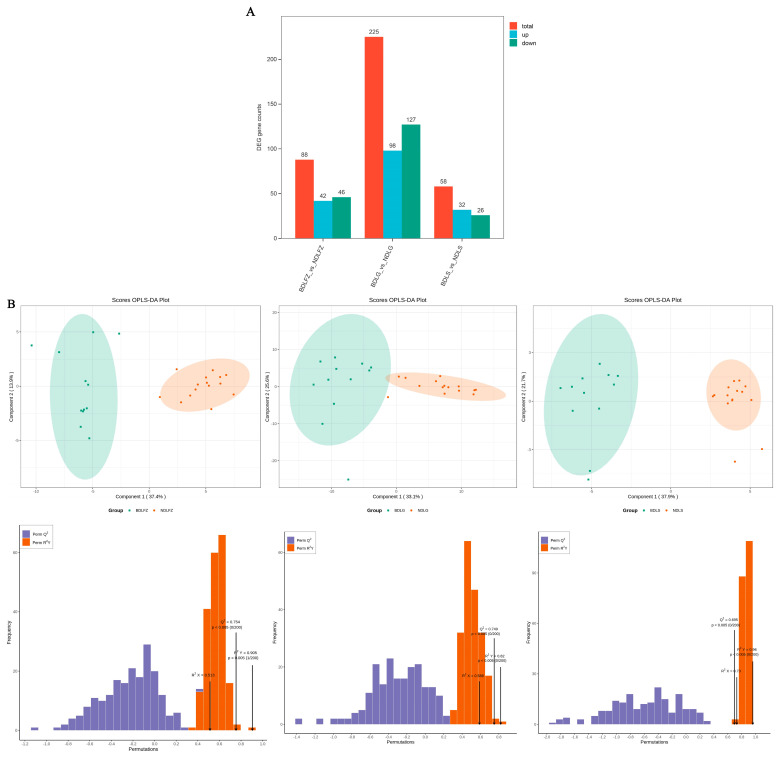

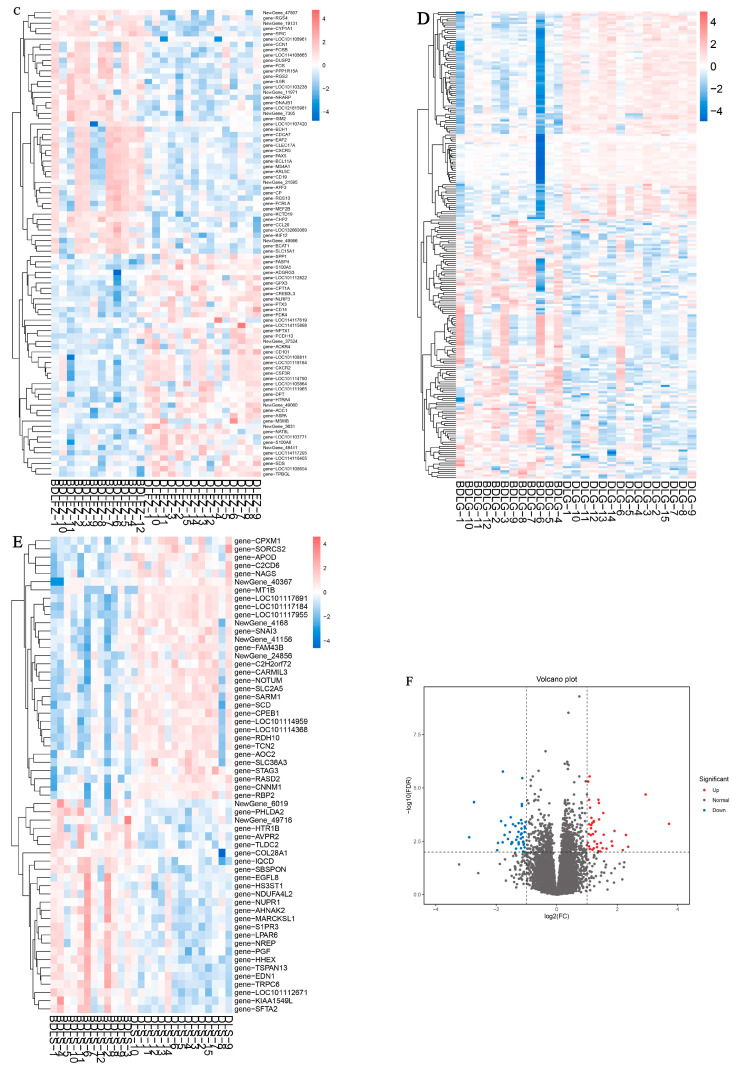

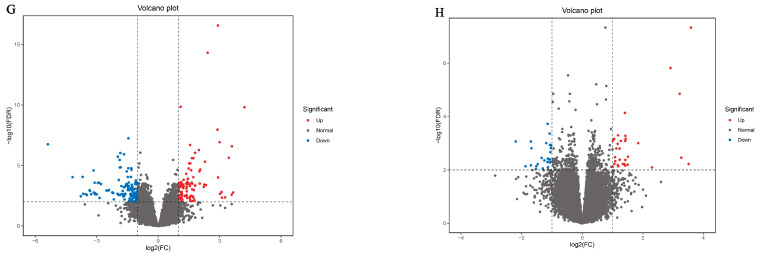
Expression profiles of differentially expressed genes between Duolang sheep in Southern Xinjiang and Duolang sheep in Northern Xinjiang. Note: Expression profiles of differential genes in Duolang sheep from southern and northern Xinjiang. (**A**): Bar chart showing the comparison of the number of differential genes among the three groups. (**B**): OPLS-DA model score plot of the three groups. (**C**–**E**): Clustered heatmaps of differential genes. (**F**–**H**): Volcano plots of differential genes in different tissues. DL-FZ: Lung of Duolang sheep from southern Xinjiang; DL-G: Liver of Duolang sheep from southern Xinjiang; DL-S: Kidney of Duolang sheep from southern Xinjiang; BDL-FZ: Lung of Duolang sheep from northern Xinjiang; BDL-G: Liver of Duolang sheep from northern Xinjiang; BDL-S: Kidney of Duolang sheep from northern Xinjiang; the same is shown below.

Through the three comparisons of BDLFZ_vs_NDLFZ, BDLG_vs_NDLG, and BDLS_vs_NDLS, 88, 225, and 58 differential genes were identified, respectively (Figure 3A). The OPLS-DA model score plot (Figure 3B) showed that the two populations were significantly clustered in relatively tight regions, with significant differences in expression patterns between Duolang sheep from northern and southern Xinjiang. In the permutation test, Q^2^ > 0.5 was used to determine the presence of significant differences between the two groups. As shown in the figure, the Q^2^ values across all tissues were greater than 0.5, confirming that the model is valid and reliable. Analysis of the differential gene clustering heatmaps (Figure 3C–E) indicated that the BDLFZ_vs_NDLFZ, BDLG_vs_NDLG, and BDLS_vs_NDLS groups all exhibited significant differences, meaning there were distinct variations in differential genes between the two sheep populations. As shown in the volcano plots (Figure 3F–H), the BDLFZ_vs_NDLFZ group contained 42 upregulated differential genes and 46 downregulated differential genes; the BDLG_vs_NDLG group had 98 upregulated differential genes and 127 downregulated differential genes; and the BDLS_vs_NDLS group included 32 upregulated differential genes and 26 downregulated differential genes.

**Figure 4:**
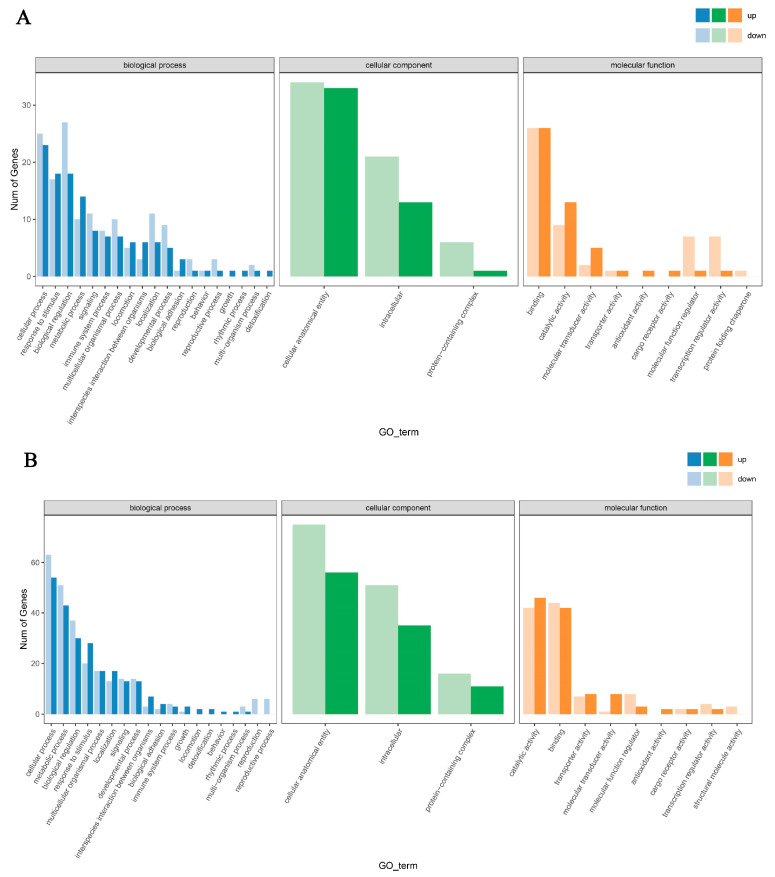

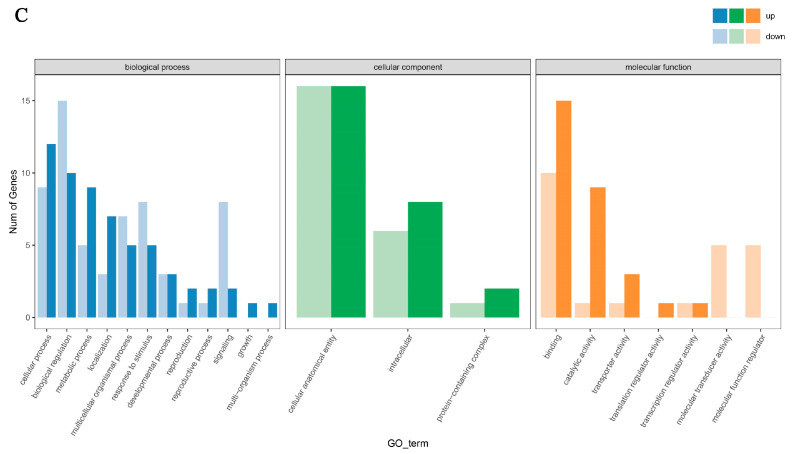
GO functional annotation analysis of differentially expressed genes among Duolang sheep from northern and southern Xinjiang. Note: (**A**–**C**): GO enrichment analysis plots in different tissues.

GO enrichment analysis was performed on differentially expressed genes identified in the BDLFZ_vs_NDLFZ, BDLG_vs_NDLG, and BDLS_vs_NDLS groups (comparing Duolang sheep from northern and southern Xinjiang), with the results shown in Figure 4. Key terms were selected using a significance threshold of *p* < 0.05. In the BDLFZ_vs_NDLFZ group, a total of 78 differentially expressed genes were enriched in 43 GO terms. Within the biological process category, the term with the most enriched genes was “cellular process”; in the cellular component category, the term with the most enriched genes was “cellular anatomical entity”; and in the molecular function category, the term with the most enriched genes was “binding”. In the BDLG_vs_NDLG group, 184 differentially expressed genes were enriched in 43 GO terms. The most highly represented term in the biological process category was “cellular process”; in the cellular component category, the most represented term was “cellular anatomical entity”; and in the molecular function category, the most represented term was “catalytic activity”. In the BDLS_vs_NDLS group, 48 differentially expressed genes were enriched in 43 GO terms. For biological processes, the term with the most enriched genes was “biological regulation”; for cellular components, the term with the most enriched genes was “cellular anatomical entity”; and for molecular functions, the term with the most enriched genes was “catalytic activity.”

**Figure 5:**
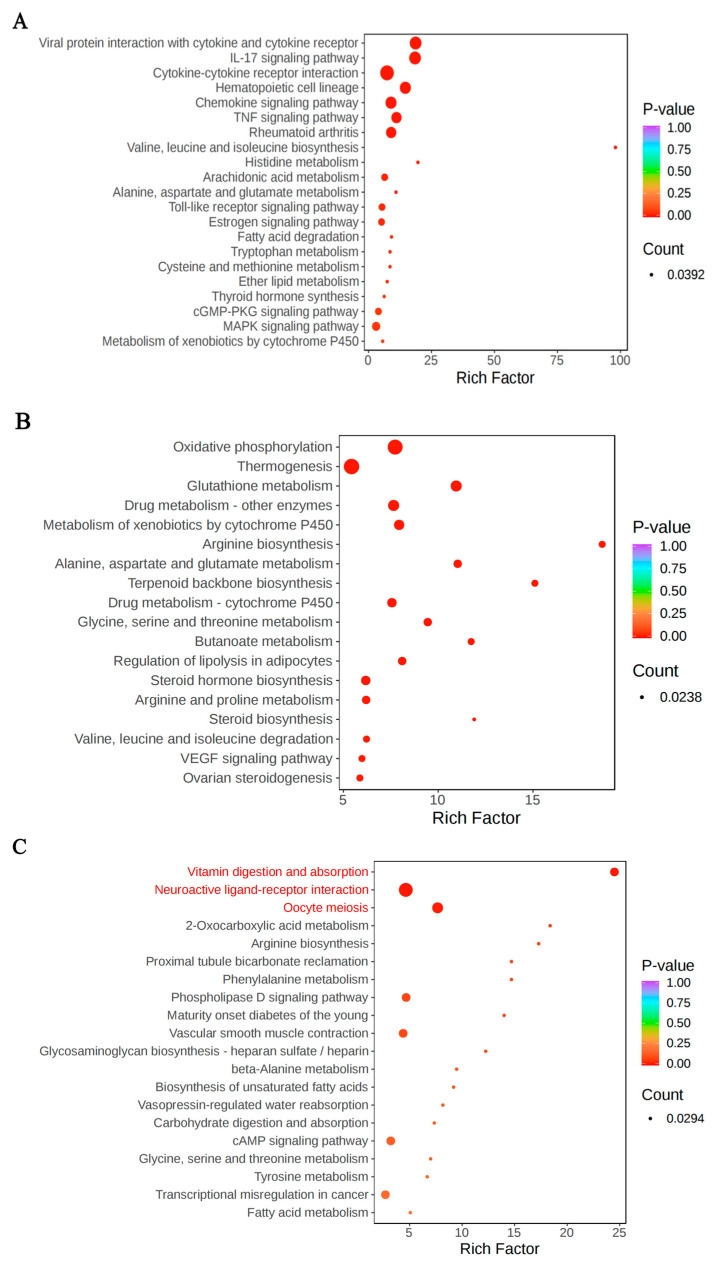

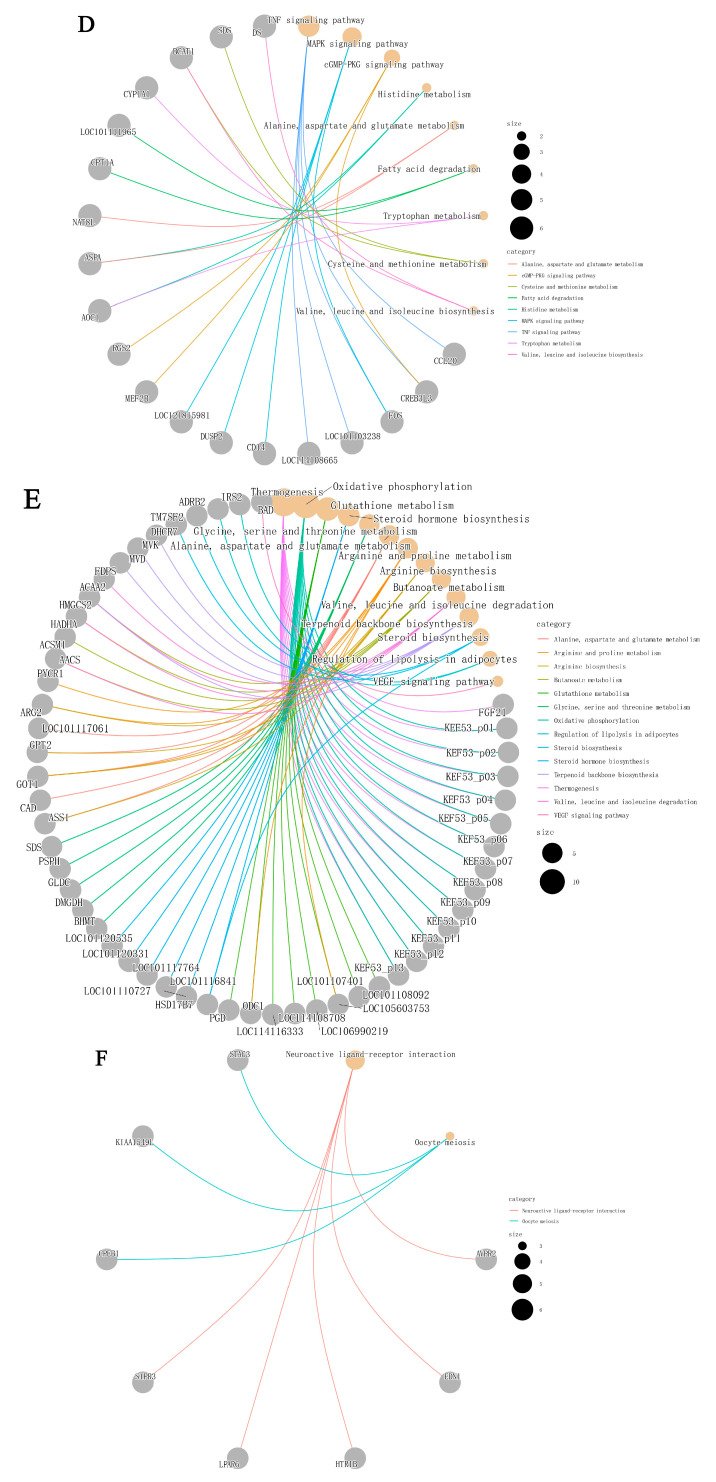
KEGG functional annotation analysis of differentially expressed genes among Duolang sheep from northern and southern Xinjiang. Note: (**A**–**C**): KEGG enrichment analysis plots in different tissues. The size of the circle represents the level of enrichment significance of the pathway (i.e., the smaller the *p*-value, the higher the enrichment significance). In subfigures (**C**), the red text indicates the key candidate pathways that are significantly expressed in each part (*p* < 0.05). (**D**–**F**): Enriched cnet plots in different tissues.

KEGG enrichment analysis was performed on differentially expressed genes identified in the BDLFZ_vs_NDLFZ, BDLG_vs_NDLG, and BDLS_vs_NDLS groups (comparing Duolang sheep from northern and southern Xinjiang), with results shown in Figure 5. The findings are summarized as follows: In the BDLFZ_vs_NDLFZ group, 37 differentially expressed genes were mapped to 21 KEGG pathways. Among these, six key candidate pathways were metabolism-related: histidine metabolism; alanine, aspartate and glutamate metabolism; tryptophan metabolism; valine, leucine and isoleucine biosynthesis; fatty acid degradation; and cysteine and methionine metabolism. In addition, three key candidate pathways were associated with environmental information processing: the cGMP-PKG signaling pathway, the MAPK signaling pathway, and the TNF signaling pathway. For the BDLG_vs_NDLG group (results shown in Figure 5), 61 differentially expressed genes were enriched in 18 KEGG pathways. Eleven of these key candidate pathways were related to metabolism: terpenoid backbone biosynthesis; glycine, serine and threonine metabolism; butanoate metabolism; steroid hormone biosynthesis; arginine and proline metabolism; steroid biosynthesis; valine, leucine and isoleucine degradation; arginine biosynthesis; glutathione metabolism; alanine, aspartate and glutamate metabolism; and oxidative phosphorylation. One key candidate pathway was linked to environmental information processing (the VEGF signaling pathway), and one was associated with organismal systems (thermogenesis). In the BDLS_vs_NDLS group, 10 differentially expressed genes were enriched in three KEGG pathways. Among these, one key candidate pathway was related to environmental information processing: neuroactive ligand–receptor interaction.

**Figure 6:**
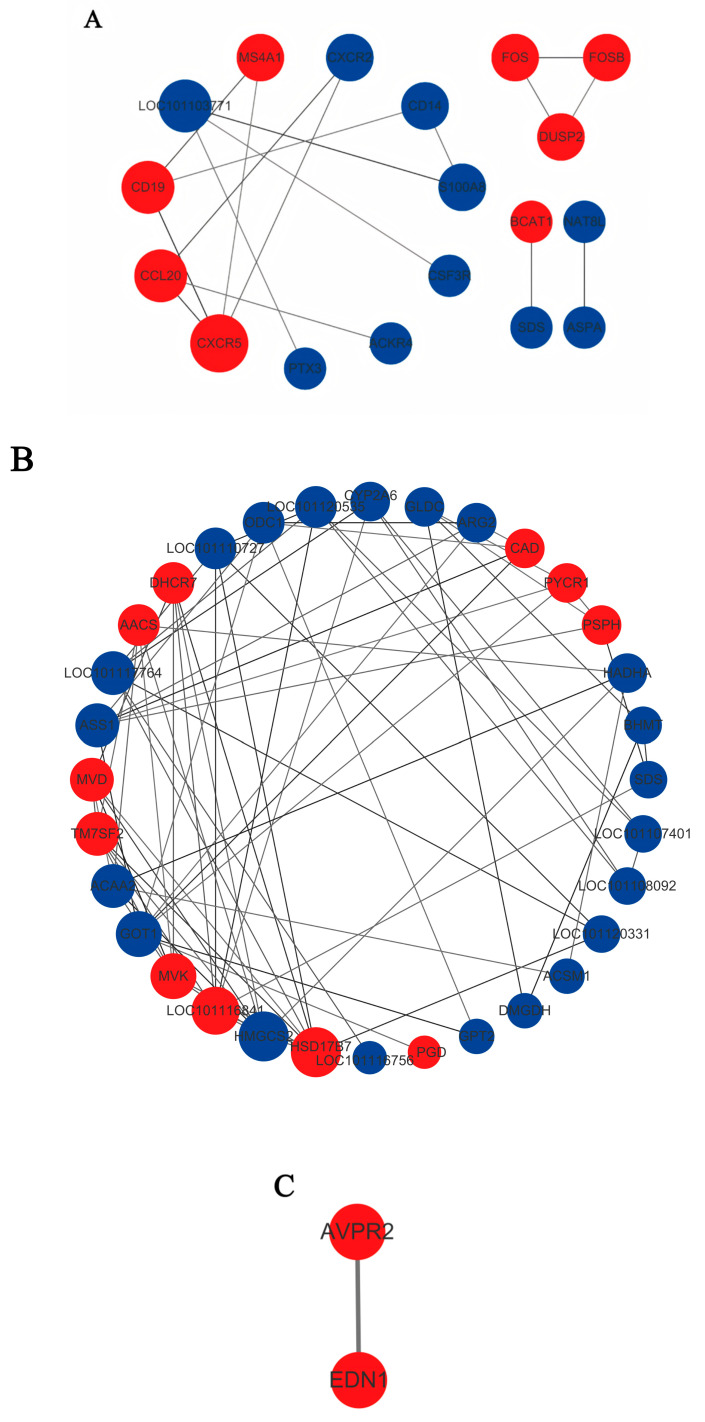
Protein–Protein Interaction Network Diagrams of Differentially Expressed Proteins. Note: (**A**–**C**): PPI network plots in different tissues. Red indicates upregulated genes/metabolites, blue indicates downregulated ones, and the circle size indicates the level of degree value (larger circles represent higher degree values).

To investigate interactions among proteins encoded by differentially expressed genes, we performed protein–protein interaction (PPI) analysis using the STRING database for each comparison group. The resulting PPI networks, which included varying numbers of differentially expressed proteins (DEPs), are shown in Figure 6. Hub genes were subsequently identified using the CytoHubba plugin in Cytoscape (version 3.9.1). The degree value of each node reflects its relative contribution to the regulation of the protein interaction network. Using a significance threshold of *p* < 0.05, the following key candidate differential genes were identified: A total of 52 core proteins encoded by differentially expressed genes were identified, the majority of which were downregulated. In the BDLFZ_vs_NDLFZ group, the proteins encoded by CD14, CCL20, BCAT1, and DUSP2 exhibited strong interaction. Among these, CCL20, BCAT1, and DUSP2 were upregulated, whereas CD14 was downregulated. In the BDLG_vs_NDLG group, strong interactions were observed among the proteins encoded by GOT1, BHMT, AACS, HADHA, and ACAA2. AACS was upregulated, while GOT1, BHMT, HADHA, and ACAA2 were downregulated. In the BDLS_vs_NDLS group, AVPR2 and EDN1 showed strong interaction, and both were upregulated.

### 3.4. Results of Integrated Analysis

**Figure 7:**
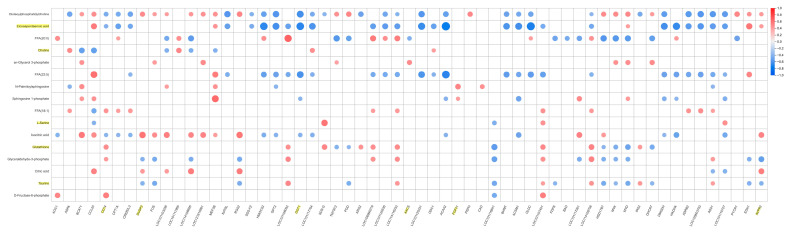
Key candidate metabolite–gene correlation matrix. In the figure, the varying sizes of the circles indicate the strength of the correlation, with red representing a positive correlation and blue representing a negative correlation.

Using FPKM values, a correlation matrix between key candidate metabolites and key candidate genes was constructed, as shown in Figure 7. The analysis revealed the following associations: EPA, a metabolite enriched in the “Biosynthesis of unsaturated fatty acids” pathway, showed a significant negative correlation with CD14 (enriched in the MAPK signaling pathway) and with GOT1 (enriched in arginine biosynthesis). In contrast, EPA exhibited a significant positive correlation with AVPR2, a gene involved in vasopressin-regulated water reabsorption. Taurine, enriched in the sulfur metabolism pathway, was significantly negatively correlated with both DUSP2 and AVPR2, both of which are associated with the MAPK signaling pathway. Additionally, CD14 showed a significant positive correlation with glutathione, a metabolite enriched in the biosynthesis of cofactors pathway.

## 4. Discussion

### 4.1. Differences in Serum Biochemical Indicators of Duolang Sheep from Different Regions

Serum biochemical indicators serve as valuable diagnostic markers for assessing animal health, as they reflect lipid mobilization, energy balance, and underlying pathophysiological processes [10]. In this study, although Duolang sheep from southern Xinjiang exhibited highly significant differences in several serum biochemical parameters compared to those from northern Xinjiang—likely due to the arid environment they inhabit—they nonetheless maintained good physiological health. GSH-PX plays a central role in detoxifying reactive oxygen species (ROS), effectively scavenging ROS and alleviating oxidative tissue damage [11]. In the present study, serum GSH-PX levels were significantly higher in southern Xinjiang Duolang sheep than in their northern counterparts (*p* < 0.01). This elevated antioxidant capacity likely represents a key adaptive response to oxidative stress induced by arid conditions, contributing to the maintenance of systemic homeostasis. IL-2 has attracted considerable attention for its role in promoting the proliferation, activation, and differentiation of key effector immune cell populations, particularly those regulated by checkpoint inhibitors [12]. As an endogenous immune-stimulating cytokine, IL-2 functions as a critical signaling molecule between immune cells, with its spatial distribution and diffusion kinetics tightly regulated to prevent aberrant or pathological immune activation [13]. In this study, serum IL-2 levels were significantly higher in southern Xinjiang Duolang sheep than in those from northern Xinjiang (*p* < 0.01), suggesting enhanced cellular immune responsiveness. This may help maintain immune homeostasis and provide more effective protection against immune dysregulation or pathogen exposure, potentially associated with arid environments. IgG is the predominant antibody isotype in serum and plays a central role in immune defense against both intracellular and extracellular pathogens [14]. Here, serum IgG levels were significantly elevated in southern Xinjiang Duolang sheep compared to the northern group (*p* < 0.01), indicating enhanced humoral immune function. This increased antibody production capacity likely reflects stronger resistance to environmental pathogens and contributes to overall health under arid conditions.

### 4.2. Analysis of Metabolite Differences

In this study, compared with Duolang sheep from the milder climate of northern Xinjiang, those raised in the arid environment of southern Xinjiang exhibited 256 upregulated and 288 downregulated metabolites in perirenal and caudal fat tissues. Prominent among the upregulated metabolites were choline, L-serine, glutathione, and taurine, whereas notably downregulated metabolites included EPA and sphingosine-1-phosphate.

Based on untargeted metabolomic analysis, significant differences were observed in the expression of metabolites involved in lipid metabolism between Duolang sheep from southern and northern Xinjiang. Metabolomic data were collected from perirenal and caudal fat tissues of sheep raised under identical feeding and management conditions in both regions. KEGG enrichment analysis identified EPA, a metabolite enriched in the “Biosynthesis of unsaturated fatty acids” pathway, as a key candidate differential metabolite in the BDLFZ_vs_NDLFZ group, with downregulated expression in the southern Xinjiang group. EPA regulates lipid metabolism by inhibiting de novo fatty acid synthesis, promoting fatty acid β-oxidation [15], and accelerating saturated fatty acid (SFA) metabolism [16]. In addition, EPA exerts beneficial physiological effects such as reducing cholesterol and triglyceride (TG) levels and increasing high-density lipoprotein cholesterol (HDL-C) [16]. These effects are mediated through downregulation of PPARγ, SREBP-1, and FAS gene expression [17] and upregulation of PPARα expression in adipocytes [18]. Studies have also shown that EPA reduces abdominal fat deposition in grass carp by promoting triglyceride hydrolysis, inducing adipocyte apoptosis, and inhibiting de novo fatty acid synthesis [15]. In the present study, downregulation of EPA in southern Xinjiang Duolang sheep suggests that, compared with their northern counterparts, arid environmental stress may enhance the inhibition of de novo fatty acid synthesis, promote fatty acid oxidation, and reduce the metabolic rate of SFAs. Reduced fat deposition may also serve as an adaptive strategy to mitigate inflammation and oxidative stress induced by arid conditions, thereby supporting overall health. Choline, enriched in the “Glycerophospholipid metabolism” pathway, was identified as another key candidate differential metabolite, with upregulated expression in the southern Xinjiang group. Choline (molecular formula: (CH_3_)_3_N(CH_2_)_2_OH) is a potent organic base and a biosynthetic precursor of the neurotransmitter acetylcholine. As a water-soluble vitamin-like substance, choline plays an essential role in fish survival and growth by participating in the synthesis and metabolism of phospholipids, amino acids, and nucleotides through methyl group donation. Its metabolic regulatory function is therefore closely linked to the supply of active methyl groups for energy metabolism [19]. Additional studies have suggested that the positive effects of choline supplementation on growth performance in fish may be related to its involvement in regulating glucose and lipid metabolism [20]. The observed upregulation of choline in southern Xinjiang Duolang sheep indicates that, under arid stress, these animals may enhance phospholipid synthesis and energy metabolism by increasing methyl donor activity. This adaptation could help alleviate oxidative stress and inflammatory responses, thereby maintaining physiological homeostasis under harsh environmental conditions. Moreover, this upregulation may contribute to the regulation of glucose and lipid metabolic pathways, optimize fatty acid utilization, reduce the metabolic burden associated with fat deposition, and ultimately improve survival adaptability and preserve health.

In the BDLWZ_vs_NDLWZ group, L-serine and glutathione—both enriched in the “Biosynthesis of cofactors” pathway—were identified as key candidate differential metabolites, with upregulated expression in the southern Xinjiang group. L-serine is an amino acid with important physiological functions. It serves as a precursor for glycine and cysteine and has been recognized as an effective antioxidant and anti-stress agent. Its antioxidant mechanisms are primarily mediated through the promotion of glutathione synthesis and related metabolic pathways. In mouse studies, Ref. [21] first demonstrated that L-serine enhances antioxidant capacity by activating the methionine cycle and promoting glutathione synthesis. As a key endogenous antioxidant molecule, glutathione biosynthesis depends on glycine and cysteine as essential substrates. By acting as a precursor for both amino acids, L-serine facilitates glutathione production, thereby strengthening the antioxidant defense system. Ref. [22] investigated broilers during the tropical dry season and found that L-serine supplementation effectively improved antioxidant capacity under combined heat and nutritional stress, consistent with the findings of [21]. Through the sequential pathway of precursor supply, enhanced glutathione synthesis, improved antioxidant capacity, and attenuated stress responses, L-serine holds significant potential for regulating antioxidant and anti-stress mechanisms in animals. In the present study, the upregulated expression of both L-serine and glutathione in southern Xinjiang Duolang sheep suggests that arid environmental conditions may induce oxidative stress, potentially affecting growth performance and health. However, the elevated levels of these metabolites likely represent an adaptive response that mitigates the negative effects of arid stress, enhances antioxidant capacity, and ultimately supports physiological homeostasis and survival under challenging conditions.

Taurine, enriched in the “sulfur metabolism” pathway, was identified as a key candidate differential metabolite with upregulated expression in the southern Xinjiang group. As one of the most abundant free amino acids in the body, taurine exhibits a broad range of biological functions, including participation in glucose and lipid metabolism, anti-inflammatory and antioxidant effects, and stabilization of cell membrane structures [23]. Supplementation with taurine has been shown to enhance antioxidant capacity, alleviate lipopolysaccharide (LPS)-induced inflammatory responses and oxidative stress [24], and reduce levels of inflammatory markers in animals [25]. Taurine also helps mitigate tissue damage caused by hyperglycemia and contributes to improved blood glucose stability [26]. Furthermore, it plays a key physiological role in maintaining and restoring redox balance by regulating endogenous antioxidants such as glutathione [27].

The upregulation of taurine in southern Xinjiang Duolang sheep suggests that, in response to arid stress, taurine enhances antioxidant defense capacity, effectively neutralizing reactive oxygen species (ROS) accumulation induced by arid conditions and thereby reducing lipid peroxidation damage to cell membranes. Its anti-inflammatory properties help alleviate tissue inflammation, while its role in stabilizing blood glucose and improving glucose and lipid metabolism supports energy homeostasis. Together, these metabolic adjustments optimize adaptive strategies, ultimately enhancing both survival resilience and production potential under arid environmental conditions.

### 4.3. Functional Analysis of Differential Genes in the Lung, Liver, and Kidney Tissues of Duolang Sheep from Different Regions

RNA-seq analysis revealed significant differences in the expression of genes associated with environmental adaptability between Duolang sheep from southern and northern Xinjiang. Transcriptomic data were collected from lung, liver, and kidney tissues of sheep raised under identical feeding and management conditions in both regions. KEGG enrichment analysis yielded the following findings:

In the BDLFZ_vs_NDLFZ group, CD14 and DUSP2—both enriched in the MAPK signaling pathway—were identified as key candidate differential genes. CD14 was downregulated, while DUSP2 was upregulated. The MAPK signaling pathway plays a critical role in regulating cellular responses to various environmental stresses, including temperature extremes, oxidative stress, pathogen infection, and drought [28]. Studies have shown that the strong adaptability of sheep to arid and high-salt environments is partly attributed to the activity of this pathway [29]. CD14 is an important pattern recognition receptor in innate immunity. It functions through multiple Toll-like receptor (TLR) signaling complexes and, upon ligand binding, induces intracellular pro-inflammatory signaling cascades [30]. DUSP2 inhibits adipogenesis by negatively regulating the mitogen-activated protein kinase (MAPK) family, a key pathway involved in cell proliferation, differentiation, and adipogenesis [31]. In the present study, the downregulation of CD14 in southern Xinjiang Duolang sheep suggests that despite exposure to arid conditions, their immune response remains appropriately regulated, helping to prevent excessive inflammation and maintain tissue health. Meanwhile, the upregulation of DUSP2 points to enhanced inhibition of adipogenesis, which may limit fat accumulation. Reduced fat deposition likely represents an adaptive strategy to mitigate inflammation and oxidative stress induced by arid environments, thereby supporting overall physiological homeostasis.

RNA-seq analysis revealed significant differences in the expression of genes associated with environmental adaptability between Duolang sheep from southern and northern Xinjiang. Transcriptomic data were collected from lung, liver, and kidney tissues of sheep raised under identical feeding and management conditions in both regions. KEGG enrichment analysis yielded the following findings: FGF21, enriched in the “Thermogenesis” pathway, was identified as a key candidate differential gene with upregulated expression in the southern Xinjiang group. As a hormone involved in regulating key metabolic pathways, FGF21 promotes fatty acid oxidation and ketogenesis, inhibits adipogenesis, and enhances glucose uptake, amino acid transport, and energy expenditure [32]. These functions support its role as a stress hormone that mobilizes energy substrates to meet the demands of energy-consuming stress responses [33]. Studies have also shown that FGF21 significantly reduces triglyceride deposition in muscle tissue of newborn piglets [34], and Ref. [35] reported that FGF21 induces reduced hepatic very low-density lipoprotein synthesis in ruminants. In the present study, the upregulation of FGF21 aligns with findings by [36] and suggests that under drought stress, southern Xinjiang Duolang sheep exhibit enhanced drought adaptability and oxidative stress resistance. The contribution of FGF21 to inhibiting adipogenesis and promoting fatty acid oxidation may help maintain physiological health under arid conditions. GOT1, enriched in the “Arginine biosynthesis” pathway, was identified as a key candidate differential gene with downregulated expression. GOT1 is an enzyme with lipolytic or lipase activity and plays an important role in maintaining cellular function under stress. Previous studies have shown that GOT1 reduces reactive oxygen species (ROS) production [37] and participates in sulfur dioxide synthesis, producing cytoprotective SO_2_ [38]. GOT1 is also involved in the biosynthesis of organic nitrogen compounds [39]. In the context of fatty acid metabolism, Ref. [40] reported that soluble aspartate transaminase (GOT1) supplies oxaloacetate under low-glucose conditions, a mechanism that may contribute to maintaining redox homeostasis. In this study, the downregulation of GOT1 in southern Xinjiang Duolang sheep suggests that arid conditions impose increased oxidative stress. Nevertheless, GOT1 may still function to reduce ROS production, regulate oxidative stress levels, maintain cellular homeostasis, and alleviate inflammation. Moreover, reduced GOT1 expression may reflect enhanced fatty acid oxidation and suppressed adipogenesis, supporting survival under arid environmental stress. AACS, enriched in the “Butanoate metabolism” pathway, was identified as a key candidate differential gene with upregulated expression. Acetoacetyl-CoA synthase (AACS) is a cytoplasmic ketone body (acetoacetate)-specific ligase widely distributed in adipogenic tissues [41]. This enzyme catalyzes the conversion of acetoacetate to acetoacetyl-CoA, thereby participating in cholesterol and fatty acid biosynthesis [41]. Ref. [42] reported that AACS is highly expressed in adipose tissue and negatively regulates subcutaneous fat deposition in pigs, with upregulation inhibiting preadipocyte differentiation. Additionally, Ref. [43] found that plasma AACS levels are positively correlated with hepatic lipid deposition. In the present study, the upregulation of AACS in southern Xinjiang Duolang sheep is consistent with these findings, suggesting that fat deposition is inhibited under arid conditions—an adaptive response that may contribute to maintaining health and physiological function in challenging environments.

PGF, enriched in the PI3K-Akt signaling pathway, was identified as a key candidate gene with upregulated expression. The PI3K-Akt pathway plays a crucial role in mediating cellular responses to drought stress, particularly in maintaining cell survival, regulating energy metabolism, and alleviating stress-induced damage. Drought conditions elevate intracellular oxidative stress, which may trigger apoptosis. Activation of the PI3K-Akt pathway counteracts this by upregulating anti-apoptotic factors, thereby promoting cell survival [44]. In mammals, including ruminants, water and feed scarcity under arid conditions further engage this pathway to regulate lipid mobilization and metabolic adaptation [45]. AVPR2, enriched in the “vasopressin-regulated water reabsorption” pathway, was identified as another key candidate gene with upregulated expression. The classical action of antidiuretic hormone (arginine vasopressin, AVP) involves binding to AVP receptor 2 (AVPR2), which triggers a cAMP-mediated cascade that promotes aquaporin 2 (AQP2) insertion into the luminal membrane of collecting ducts, thereby stimulating water reabsorption [46]. Studies have shown that the mammalian kidney itself can produce AVP, supplying a local source for AVPR2 activation via paracrine signaling and further enhancing water reabsorption through AQP2 [47,48]. Additionally, AVPR2 has been implicated in regulating stress responses [49]. In this study, the upregulation of AVPR2 in southern Xinjiang Duolang sheep suggests enhanced renal water reabsorption capacity, enabling these animals to maintain fluid balance and normal physiological function under extreme arid conditions. It is also plausible that AVPR2 contributes to antioxidant stress regulation, thereby supporting overall health and adaptation in harsh environments.

### 4.4. Integrated Analysis

Under oxidative stress, mitochondrial function is often compromised. Fatty acid metabolism—particularly β-oxidation—plays a central role in cellular energy homeostasis [50]. By integrating lipid metabolomics, transcriptomics, and serum biochemical analyses, this study elucidated the core adaptive mechanisms employed by southern Xinjiang Duolang sheep in response to drought stress. Lipid metabolomics revealed that Duolang sheep enhance fatty acid β-oxidation through the regulation of key metabolites, including eicosapentaenoic acid (EPA), choline, L-serine, glutathione, and taurine. This metabolic adjustment enables efficient mobilization and oxidation of stored lipids under conditions of water scarcity and potential nutrient limitation, thereby supporting ATP production and maintaining cellular energy balance. Complementary transcriptomic and serum biochemical analyses showed that Duolang sheep upregulated the expression of genes such as DUSP2, FGF21, AACS, PGF, and AVPR2, while downregulating CD14 and GOT1. These expression changes facilitate the clearance of reactive oxygen species (ROS) accumulated under drought conditions, protect cellular structures from oxidative damage, and enhance immune function. They also help attenuate pro-inflammatory signaling and reduce disease susceptibility, thereby strengthening the animals’ resilience under environmental stress. Together, these multi-omics findings demonstrate that drought adaptation in Duolang sheep involves coordinated adjustments in lipid metabolism, antioxidant defense, and immune regulation—all contributing to maintained physiological integrity and enhanced survival in arid environments.

The core adaptive mechanisms of Duolang sheep in response to drought stress involve two complementary aspects. On the one hand, they reconfigure energy metabolic pathways to maintain an adequate energy supply under stress. On the other hand, they mount a robust antioxidant and immune defense to effectively counteract stressors—such as disease risk—potentially intensified by arid environments. Together, these strategies synergistically enhance their overall adaptability to drought conditions.

## 5. Conclusions

Differences in antioxidant and immune indicators suggest that adapting to arid environments involves enhanced oxidative stress resistance and immune responsiveness. Key candidate metabolites, such as EPA, enriched in the “Biosynthesis of unsaturated fatty acids” pathway, exhibit differential abundance between southern and northern Xinjiang Duolang sheep and are associated with suppressed fat synthesis. This points to a metabolic shift toward enhanced fatty acid β-oxidation under arid conditions, helping maintain energy homeostasis. Similarly, key candidate genes, including FGF21, enriched in the “Thermogenesis” pathway, show significant expression differences between the two groups. These genes participate in lipid metabolism by promoting fatty acid oxidation and reducing triglyceride accumulation, thereby supporting the adaptation of southern Xinjiang Duolang sheep to their arid environment.

## Figures and Tables

**Table 1:** Blood biochemical parameter test results of Duolang sheep from southern and northern Xinjiang.

Measurement Parameters	NDL	BDL
MDA (nmol/mL)	4.86 ± 1.84 ^a^	5.17 ± 1.46 ^a^
GSH-PX (U/mL)	55.86 ± 17.23 ^A^	40.60 ± 7.80 ^B^
CAT (U/mL)	1.50 ± 1.28 ^a^	2.14 ± 1.67 ^a^
T-AOC (mmol/L)	0.74 ± 0.07 ^a^	0.79 ± 0.09 ^a^
LDH (U/L)	2664.54 ± 289.06 ^a^	2722.61 ± 404.53 ^a^
GLU (mmol/L)	4.19 ± 1.14 ^a^	3.77 ± 0.85 ^a^
Ca (mmol/L)	1.75 ± 0.12 ^a^	1.86 ± 0.16 ^a^
IgG (mg/mL)	9.65 ± 1.44 ^A^	7.35 ± 1.81 ^B^
IL-2 (ng/L)	44.68 ± 9.75 ^A^	33.04 ± 14.03 ^B^

Note: Different lowercase superscript letters in the same row indicate a significant difference (*p* < 0.05); different uppercase superscript letters indicate an extremely significant difference (*p* < 0.01); the same superscript letters or no superscript letters indicate no significant difference (*p* > 0.05). NDL: Duolang sheep from southern Xinjiang, BDL: Duolang Sheep from northern Xinjiang; the same below.

## Data Availability

All data generated during this study have been submitted to the BioProject database at NCBI under the BioProject ID: PRJNA1370875.

## Abbreviations

The following abbreviations are used in this manuscript:

GSH-Px	glutathione peroxidase
IL-2	interleukin-2
IgG	immunoglobulin G
EPA	eicosapentaenoic acid
RNA-seq	reference-based transcriptomics
RIN	RNA integrity
UHPLC	ultra-high performance liquid chromatography
PE150	paired-end 150
GO	gene ontology
KEGG	Kyoto Encyclopedia of Genes and Genomes
PPI	protein–protein interaction
DEP	differentially expressed protein
ROS	reactive oxygen species
SFA	saturated fatty acid
TG	triglyceride
HDL-C	high-density lipoprotein cholesterol
LPS	lipopolysaccharide
TLR	Toll-like receptor
MAPK	mitogen-activated protein kinase
AACS	Acetoacetyl-CoA synthase
AQP	aquaporin
AVP	antidiuretic hormone
ATP	adenosine triphosphate

## Appendix A

**Table A1:** ELISA Detection Procedures.

(1) Plate equilibration: Remove the required microplates and equilibrate at room temperature for 20 min; return unused plates to 4 °C storage.
(2) Standard setup: Add 50 μL of standard solutions at different concentrations to the designated standard wells.
(3) Sample preparation: Add 10 μL of the test sample to sample wells, followed by 40 μL of sample diluent, and mix gently.
(4) HRP-conjugated antibody addition: Add 100 μL of horseradish peroxidase (HRP)-conjugated detection antibody to each well. Seal the plate with adhesive film and incubate at 37 °C for 1 h in a thermostatic incubator.
(5) Plate washing: Discard the supernatant, fill each well with washing buffer, let stand for 60 s, then flick off the liquid. Repeat washing 5 times and pat the plate dry on absorbent paper.
(6) Substrate addition: Add 50 μL of Substrate A and 50 μL of Substrate B to each well. Incubate at 37 °C in the dark for 15 min.
(7) Reaction termination: Add 50 μL of stop solution to each well. Measure the optical density (OD) at 450 nm using a microplate reader within 15 min.
(8) Standard curve construction: Plot a linear regression curve of standard concentration versus OD value in Excel. Calculate sample concentrations using the derived curve equation.
